# A non-randomized trial of conversion from ciclosporin and tacrolimus to tacrolimus MR4 in stable long-term kidney transplant recipients: Graft function and influences of *ABCB1* genotypes

**DOI:** 10.1371/journal.pone.0218709

**Published:** 2019-07-02

**Authors:** Markus Riegersperger, Max Plischke, Anita Jallitsch-Halper, Corinna Steinhauser, Manuela Födinger, Wolfgang C. Winkelmayer, Daniela Dunkler, Gere Sunder-Plassmann

**Affiliations:** 1 Division of Nephrology and Dialysis, Department of Medicine III, Medical University of Vienna, Austria, Europe; 2 Department of Medicine IV with Cardiology, Hospital Hietzing, Vienna, Austria, Europe; 3 Institute of Laboratory Diagnostics, Kaiser Franz Josef Hospital, Vienna, Austria, Europe; 4 Medical Faculty, Sigmund Freud Private University, Vienna, Austria, Europe; 5 Baylor College of Medicine, Section of Nephrology, Department of Medicine, Baylor Clinic, Houston, Texas, United States of America; 6 Center for Medical Statistics, Informatics and Intelligent Systems, Medical University of Vienna, Austria, Europe; Medizinische Universitat Graz, AUSTRIA

## Abstract

In this non-randomized extension study of a randomized controlled trial we converted 87 stable long-term kidney transplant recipients (KTR) from either ciclosporin (CSA, n = 28) or tacrolimus (TAC, n = 59) to TAC modified release (TAC MR4) to study the characteristics of TAC trough levels after conversion with the primary endpoint graft function after 12 months. TAC MR4 consumption was calculated by level-to-dose ([ng/mL]/[mg/d]) and concentration-to-dose ([mg/kg])/d) ratios. Influences of *ABCB1* single nucleotide polymorphisms (2677G>T/A, 1236C>T, 3435C>T) on TAC metabolism were studied. Graft function of KTR converted from CSA to TAC MR4 significantly declined over 12 months, and remained unchanged after conversion from TAC to TAC MR4. Conversion from CSA to TAC MR4 resulted in supra therapeutic- and conversion from TAC to TAC MR4 in low trough levels. We could not find associations of *ABCB1* genotypes and TAC MR4 trough levels. Adverse events and errors with TAC/TAC MR4 intake were common. In stable long-term KTR conversion from TAC to TAC MR4 is feasible. For conversion from CSA we suggest a rate of 1:40 for a rough estimation of TAC MR4 target doses.

**Trial registration:**

PEP Study: Ethics committee N° 393/2004, EudraCT 2004-004209-98.

PEP-X Study: Ethics committee amendment application N° 154/01/2008.

ClinicalTrials.gov NCT03751332.

## Introduction

The immunosuppressant tacrolimus (TAC) is a standard medication for the prevention of rejection in organ transplantation. Organ dysfunction, rejection and graft-loss due to non-adherence to immunosuppressive agents are observed in as many as 20% of kidney transplant recipients (KTR) [1]. Modified release tacrolimus (TAC MR4) with once-daily (QD) dosing was introduced to the European market in 2007 with the intention to improve drug adherence. TAC MR4 has a t_max_ of 2 to 2.5 hours, a similar t_1/2_, and a reduced c_max_ compared to TAC twice-daily (BID) [2]. The area under the concentration-time curve (AUC_0-24_) of TAC MR4 in KTR has conversely been reported to be equal [2] and higher [3]. Higher inter-patient AUC-variability [4] and lower inter-patient C_0_ levels, as well as a lower intra-patient 24-hour variability of drug exposure after conversion from TAC to TAC MR4 have been observed [5–7]. Conversion with a 1:1 ratio [8, 9] resulted in comparable trough levels, equivalent drug requirements and unchanged graft function in stable KTR [10]. Equivalent safety, efficacy, dosing and trough levels after conversion were suggested in long-term and *de novo* KTR [11–15]. However, reduced TAC MR4 C_0_ levels [5, 16–18] and dose increments [19, 20] as well as sustained low C_0_ levels despite dose increments [21–24] have been observed. Mutations in the multi-drug resistance (MDR) gene 1 (gene symbol *ABCB1*) have been reported to influence TAC dose-adjusted trough levels [25], level to dose (L/D) [16] and concentration to dose (C/D) ratios [26], mostly observed for the *ABCB1* 2677G>T/A [26–29], *ABCB1* 1236C>T [28, 29], and *ABCB1* 3435C>T [30–34] genotypes.

Data on the impact of *ABCB1* genotypes on TAC MR4 metabolism are scarce. We sought to examine TAC MR4 trough levels after conversion from either long-term ciclosporin (CSA) or TAC, to analyze the influence of *ABCB1* polymorphisms on L/D and C/D ratios, and to investigate effects of TAC MR4 on graft function in a large cohort of stable long-term KTR in eastern Austria. This study is the first to describe the local distribution of these polymorphisms, and to analyze their influences on TAC MR4 metabolism.

## Methods

### Study design

This is a non-randomized uncontrolled open-label prospective 12 month cohort extension study of the randomized controlled trial (RCT) “The Vienna Prograf and Endothelial Progenitor Cell (PEP) Study” (local ethics committee N° 393/2004, EudraCT 2004-004209-98). PEP was a RCT to investigate the effects of TAC on endothelial progenitor cells in 148 KTR [35]. The extension study PEP-X was approved by the Ethics Committee of the Medical University of Vienna (MUV) as an amendment to PEP on February 7^th^ 2008 with the application N° 154/01/2008 and registered with the governmental health agency. Since the extended release of studies within international registries conforms with today’s standard, the study was registered *post-hoc* with ClinicalTrials.gov (NCT03751332) after the study had already been completed. The authors confirm that there are no further studies related to this trial. The study has been conducted according to the “World Medical Associations Declaration of Helsinki Statement of Ethical Principles for Medical Research Involving Human Subjects”, and the “Declaration of Istanbul on Organ Trafficking and Transplant Tourism” [36]. The authors of this manuscript are aware of, and fully comply with, the “Best Practice Guidelines of the Committee on Publication Ethics (COPE)” [37]. We are reporting these findings according to the „Strengthening the reporting of genetic association studies (STREGA)”initiative [38], the „Improving the reporting quality of nonrandomized evaluations of behavioral and public health interventions: the TREND statement” [39], and, since this trial is an extension of an RCT, partly to the “CONSORT 2010 Statement: updated guidelines for reporting parallel group randomized trials” [40], respectively. One hundred forty-one patients completed the PEP trial. Two individuals out of the ITT population of 141 patients from the preceding PEP trial were deemed unsuitable at the investigators discretion, due to issues to properly follow the protocol of PEP. Hence, 139 patients (95 TAC, 44 CSA) who had completed the PEP trial were deemed eligible for inclusion. This study was conducted at the Outpatient Service of the Division of Nephrology and Dialysis, Department of Medicine III, Medical University of Vienna. Recruitment started with March 2008, the first individual was included on March 21^st^ 2008. The last on July 11^th^ 2009, and finished the study on July 7^th^ 2010. Every participant gave verbal and written informed consent prior to inclusion, and was to be converted from either CSA or TAC with or without mycophenolate mofetil (MMF) and prednisolone (PRED) to TAC MR4. Conversion from TAC to TAC MR4 was carried out in a 1:1 ratio, and conversion from CSA to TAC MR4 in a dose of 0.1 mg/kg bodyweight. To achieve maximal absorption rates patients were advised to take TAC MR4 in the morning on an empty stomach, or at least 1 hour before, or 2–3 hours after breakfast [9]. The dose was adjusted to attain TAC MR4 whole blood trough concentrations of 5–8 ng/mL. Study visits were at baseline (BL; time point 0), and in weeks 1, 2, 4, 12 and month 12 after conversion, which was the end of the study (EOS). In-between study visits patients followed the practice pattern of our transplant center, with clinical check-ups roughly every 6 to 12 weeks. The primary endpoint was graft function, as reflected in the estimated glomerular filtration rate (eGFR), after 12 months. The values compiled at visits in weeks 1 to 12 served safety purposes only, since adaptations of TAC MR4 were to be expected after conversion from either CSA or TAC. Also, we collected the eGFR from the last two clinical visits prior to inclusion, which were conducted roughly quarterly (-6 and -3 months). We assessed baseline demographic patient characteristics, serum creatinine, TAC MR4 dose and trough levels, as well as blood pressure, height, weight and adverse events, and observed influences of the *ABCB1* 2677G>T/A, *ABCB1* 1236C>T, and *ABCB1* 3435C>T genotypes. Patient reported outcomes were collected at each visit and at the end of the study by chart review. Known common adverse events of TAC were collected at each visit by standardized questionnaires within the paper case report form.

### Laboratory methods

All standard laboratory analyses and genotyping procedures were performed at the Clinical Institute of Laboratory Medicine at the Medical University of Vienna. The eGFR was calculated using the 4-variable modification of diet in renal disease (MDRD) prediction equation [41]. TAC trough levels were analyzed from ethylene-diaminetetra-acetic-acid anticoagulated fresh whole blood samples by chemiluminiscent microparticle immunoassay, Abbott Laboratories, Abbott Park, IL, USA. DNA extraction was performed manually from blood samples with the QIAamp DSP DNA Blood Mini Kit (QIAGEN GmbH, Hilden, Germany). Polymerase chain reaction (PCR) was carried out in the primer systems *ABCB1* P 6 F/R and P 11 F/R, and genotyping performed according to published protocols [42].

### Statistical methods

Categorical data are presented as frequency (n) and percentage (%), continuous data as mean ± standard deviation (SD) and median and interquartile range (IQR), respectively. To calculate the daily consumption of TAC MR4 after conversion from CSA and TAC we computed the level-to-dose (L/D) ([ng/mL]/[mg/d]) [16] and the concentration-to-dose (C/D) ratio ([mg/kg])/d) [26]. Between-group comparisons were performed by Wilcoxon-Mann-Whitney tests, and Δ eGFR was calculated with the Wilcoxon rank sum test with continuity correction. Friedman tests were used to test for changes within groups over time (baseline to end of study). To test for Hardy-Weinberg Equilibrium (HWE) the Chi-Square (χ^2^) test was used. Data for the analysis of this study were entered manually in Excel worksheets, Microsoft Corporation, Redmond, WA, USA. Correct data entry was independently verified by two study physicians, and corrected if necessary, before entering the final statistical analyses, performed with SPSS Version 21.0, IBM Corporation, Armonk, NY, USA. A probability (P) value of less than 0.05 was considered statistically significant.

## Results

**Fig 1:**
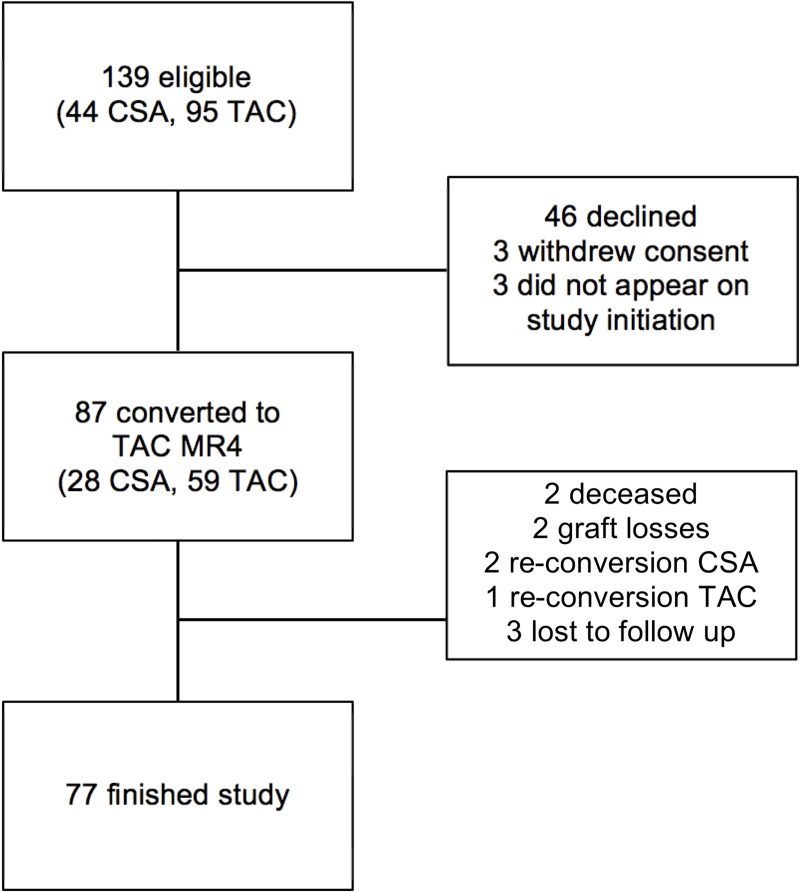
Patient disposition of the Vienna prograf and endothelial progenitor cell extension study (PEP-X). CSA = ciclosporin, TAC = tacrolimus, TAC MR4 = tacrolimus modified release.

The patient disposition is illustrated in Fig 1. Forty-six declined participation, 93 patients (63 TAC, 30 CSA) gave written informed consent for study participation. Three individuals withdrew the consent before baseline, 3 missed the initial study visit at baseline and could not be included, hence a total of 87 (65 male; 59 TAC, 28 CSA) KTR were converted from CSA or TAC to TAC MR4.

Of the 87 KTR, 4 received calcineurin inhibitor (CNI) monotherapy (2 TAC, 2 CSA), 25 CNI (20 TAC, 5 CSA) with MMF, 16 CNI (8 TAC, 8 CSA) with PRED, and 42 CNI (29 TAC, 13 CSA) with MMF and PRED. None of the patients received medication with major effects on CNI trough levels. All 87 individuals who had a single exposure with TAC MR4 were included in the intention to treat (ITT) analysis. The median age was 57.5 (49.8–67.7) years. The median time since transplantation was 9.3 (5.8–15.4) years. In the ITT population, the underlying cause of renal failure was autosomal-dominant polycystic kidney disease in 12, diabetic nephropathy in 6, glomerular disease in 26, tubule-interstitial disease in 10, vascular disease in 8, any other in 5, and unknown in 20 individuals. Six patients had a living unrelated donor, 7 a living related donor-, and 73 a deceased donor graft. Donor-specific documentation was unavailable for 1 patient. Seventy-seven KTR completed the study (2 KTR were re-converted to CSA, 1 was re-converted to TAC, there were 2 deaths and 2 graft-losses, and 3 patients were lost to follow-up). The median eGFR of the ITT population was 47.3 (39.1–60) mL/min per 1.73m^2^ at baseline and 48.3 (38–59.2) mL/min per 1.73m^2^ at month 12 (Table 1).

**Table 1:** Patient characteristics of 87 kidney transplant recipients converted from either ciclosporin or tacrolimus to modified release tacrolimus.

		PEP-X ITT n = 87	BL CSA n = 28	BL TAC n = 59
**Age**, years		57.5 (49.8–67.7)	64 (52.3–69.4)	55.8 (48.3–65.9)
**Weight**, kg		74 (67–82)	75 (71–88)	73 (65–82)
**Height**, cm		170 (167–178)	173 (169–181)	170 (165–176)
**eGFR**, mL/min per 1.73m^2^		47.3 (39.1–60)	43.5 (30.3–56.2)	49.7 (41.7–62.2)
**Time since transplantation**, years		9.3 (5.8–15.4)	11.6 (7.9–16.5)	9.2 (5.4–12.8)
**Sex**		65 male (75%)	25 male (90%)	40 male (76%)
**Ethnicity**	Caucasian	85 (98%)	27 (97%)	58 (98%)
	African	2 (2%)	1 (3%)	1 (2%)
	Asian	0	0	0
**Renal disease**	Cystic	12 (14%)	4 (14%)	8 (13%)
	Diabetic	5 (6%)	1 (3%)	4 (7%)
	Glomerular	26 (30%)	9 (33%)	17 (29%)
	Tubulointerstitial	10 (12%)	3 (11%)	7 (12%)
	Vascular	8 (9.2%)	4 (14%)	4 (7%)
	Other	5 (6%)	1 (3%)	4 (7%)
	Unknown	21 (24%)	6 (22%)	15 (25%)
**Type of donor**	Living unrelated	7 (8%)	2 (7%)	5 (8%)
	Living related	8 (9%)	3 (11%)	5 (8%)
	Deceased	72 (83%)	23 (82%)	49 (84%)
**Diabetes mellitus**	Type I	1 (1%)	0	1 (1%)
	Type II	14 (16%)	1 (3%)	13 (22%)
**Biopsy**		6 (7%)	2 (7%)	4 (7%)

PEP-X = Prograf and endothelial progenitor cell extension study, ITT = intention to treat, BL = baseline, eGFR = estimated glomerular filtration rate, CNI = calcineurin-inhibitor, CSA = ciclosporin, TAC = tacrolimus. Data are given as median and interquartile range, and as frequency and percentage, where appropriate.

After conversion from CSA to TAC MR4 the eGFR significantly declined over 12 months (43.5 [30.3–56.2] mL/min per 1.73m^2^ at baseline to 40.5 [33.7–57.3] mL/min per 1.73m^2^; p = 0.003). After conversion from TAC to TAC MR4 the eGFR remained stable (49.7 [41.7–62.2] mL/min per 1.73m^2^ at baseline and 51.3 [40.6–53.6] mL/min per 1.73m^2^ at month 12) (Tables 1 and 2). The change of the renal graft function over the study period in both conversion groups did not show any significant difference (Δ eGFR [median and 1^st^/3^rd^ quartile] CSA to TAC MR4–2.3 [-6.3/-0.4] mL/min per 1.73m^2^; TAC to TAC MR4–0.18 [-4.1/-0.8] mL/min per 1.73m^2^; p = 0.12) (Table 2).

**Table 2:** Glomerular filtration rate and median change of eGFR of 87 kidney transplant recipients after conversion from either ciclosporin (n = 28) or tacrolimus (n = 59) to tacrolimus modified release over 12 months.

	eGFR, mL/min per 1.73m^2^			
	Type of calcineurin inhibitor		Type of calcineurin inhibitor	
Point in time	CSA to TAC MR4 (n = 28)		TAC to TAC MR4 (n = 59)	
Baseline PEP-X	43.5 (30.3–56.2)	P = 0.003	49.7 (41.7–62.2)	P = 0.130
Month 12 PEP-X	40.5 (33.7–57.3)		51.3 (40.6–53.8)	
***Δ eGFR*** *(median and 1*^*st*^*/3*^*rd*^ *quartile)*, *mL/min per 1*.*73m*^*2*^	*-2*.*3* *(-6*.*3/-0*.*4)*		*-0*.*18* *(-4*.*1/-0*.*8)*	
	*P = 0*.*12*			

PEP-X = Prograf and endothelial progenitor cell extension study, CSA = ciclosporin, TAC = tacrolimus, TAC MR4 = tacrolimus modified release, eGFR = estimated glomerular filtration rate, P = probability. Data are presented as median and interquartile range.

Sixty-six (76%) patients experienced adverse events. Ten (36%) patients converted from CSA and 18 (31%) converted from TAC to TAC MR4 suffered from gastrointestinal symptoms. Pruritus was observed in 6 (21%) patients converted from CSA. Three (11%) patients in the CSA to TAC MR4 and 16 (27%) in the TAC to TAC MR4 group suffered from infections. As reported earlier, errors with TAC/TAC MR4 intake occurred [43]. Almost 10% experienced difficulties to properly follow the prescription (Table 3).

**Table 3:** Adverse events of 87 kidney transplant recipients after conversion from either ciclosporin (n = 28) or tacrolimus (n = 59) to modified release tacrolimus over 12 months.

	CSA to TAC MR4 (n = 28)	TAC to TAC MR4 (n = 59)
**Adverse events**	23 (82%)	43 (73%)
Diarrhea	9 (32%)	12 (20%)
Pruritus	4 (14%)	0
Fatigue	4 (14%)	0
Infections	3 (11%)	14 (24%)
Graft loss	3 (11%)	1 (1.7%)
Malignancy	0	2 (3.4%)
Vertigo	2 (7%)	1 (1.7%)
Erroneous intake	1 (3.5%)	4 (6.8%)

CSA = ciclosporin A, TAC = tacrolimus, TAC MR4 = tacrolimus modified release. Patient reported outcomes and known common adverse events of TAC were collected at each visit by standardized questionnaires within the paper case report form.

TAC MR4 was confused with TAC, and despite written guidance taken twice daily. Instead of TAC MR4, TAC was taken once daily. One patient was prescribed TAC MR4, but was handed out TAC at the pharmacy. The general practitioner of 1 patient converted from TAC MR4 QD to TAC QD. Two males had to be re-converted to CSA due to unbearable pruritus and gastrointestinal symptoms, which both subsided after re-conversion. One female patient with short bowel syndrome requested to be re-converted to TAC due to severe diarrhea which she thought had worsened after conversion to TAC MR4. However, this severe adverse effect remained. Ten individuals terminated the study prematurely. Of those, 2 men died (1 suffered simultaneous malignancies of lung and urinary bladder 11 months after conversion, 1 died of unknown cause 4 months after conversion). Two suffered a graft loss (1 with membranous-proliferative glomerulonephritis 2 months after conversion, 1 of unknown cause 3 months after conversion), and 3 patients (2 males) were lost to follow-up. One female developed a metastasized urothelial malignancy 6 months after conversion. We performed graft biopsies in 6 patients with acute renal failure and *de novo* proteinuria, respectively, with 1 case of borderline acute graft rejection, 1 acute vascular graft rejection Banff I, 1 membranous-proliferative glomerulonephritis, and 3 grafts with interstitial fibrosis and tubular atrophy.

**Fig 2:**
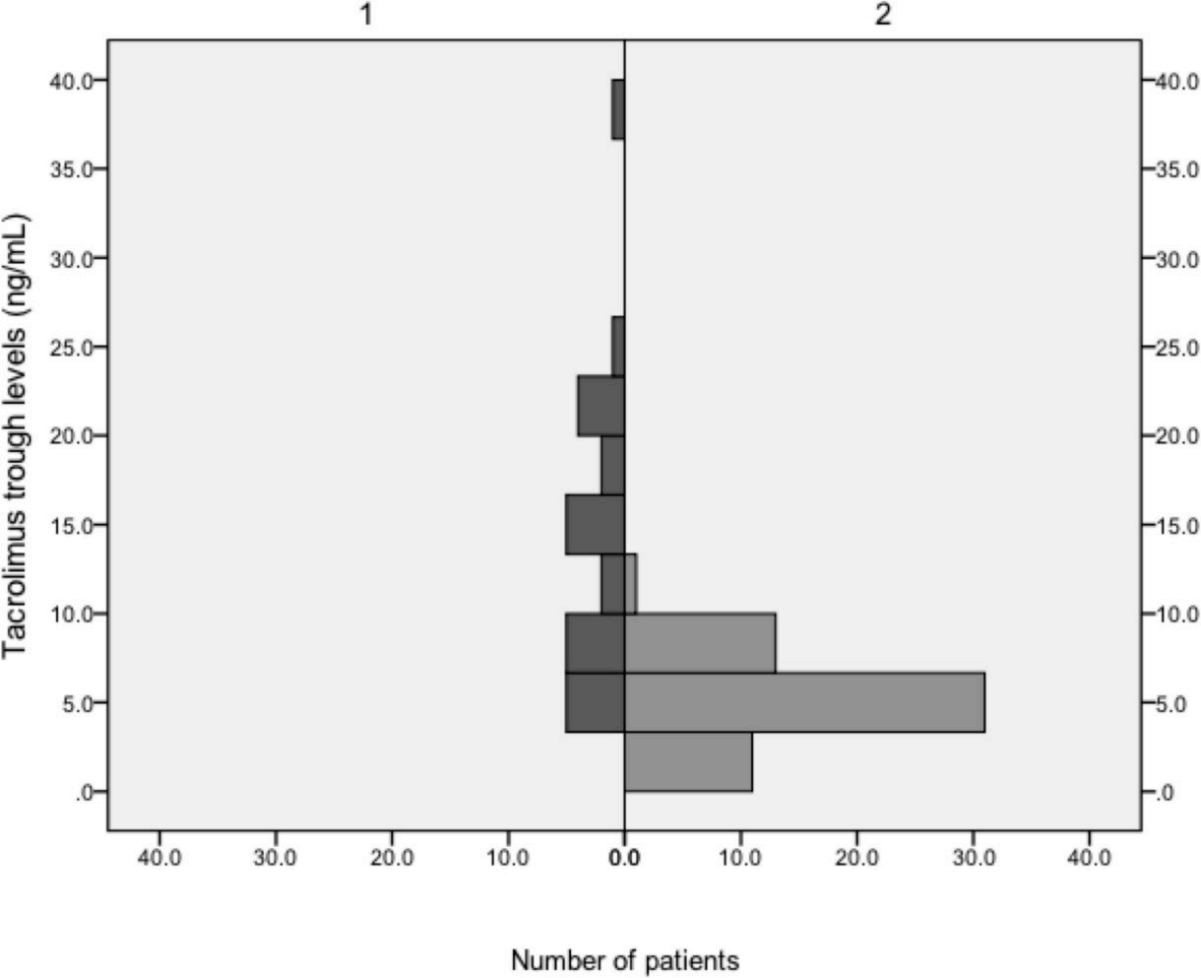
Median tacrolimus trough levels of 87 patients converted from ciclosporin and immediate release tacrolimus to modified release tacrolimus in the first week after conversion (intention to treat population). 1/dark bars = conversion from ciclosporin-based immunosuppression (n = 25; the conversion was performed in 28 individuals, 1 had taken TAC immediately before blood sampling and was excluded from the analysis, 1 failed to appear for the scheduled appointment, and 1 re-converted himself to CSA within the first days of TAC MR4 exposure), 2/light bars = conversion from tacrolimus-based immunosuppression (n = 59). Please note that scale of ordinate and abscissa differs from Fig 3.

**Fig 3:**
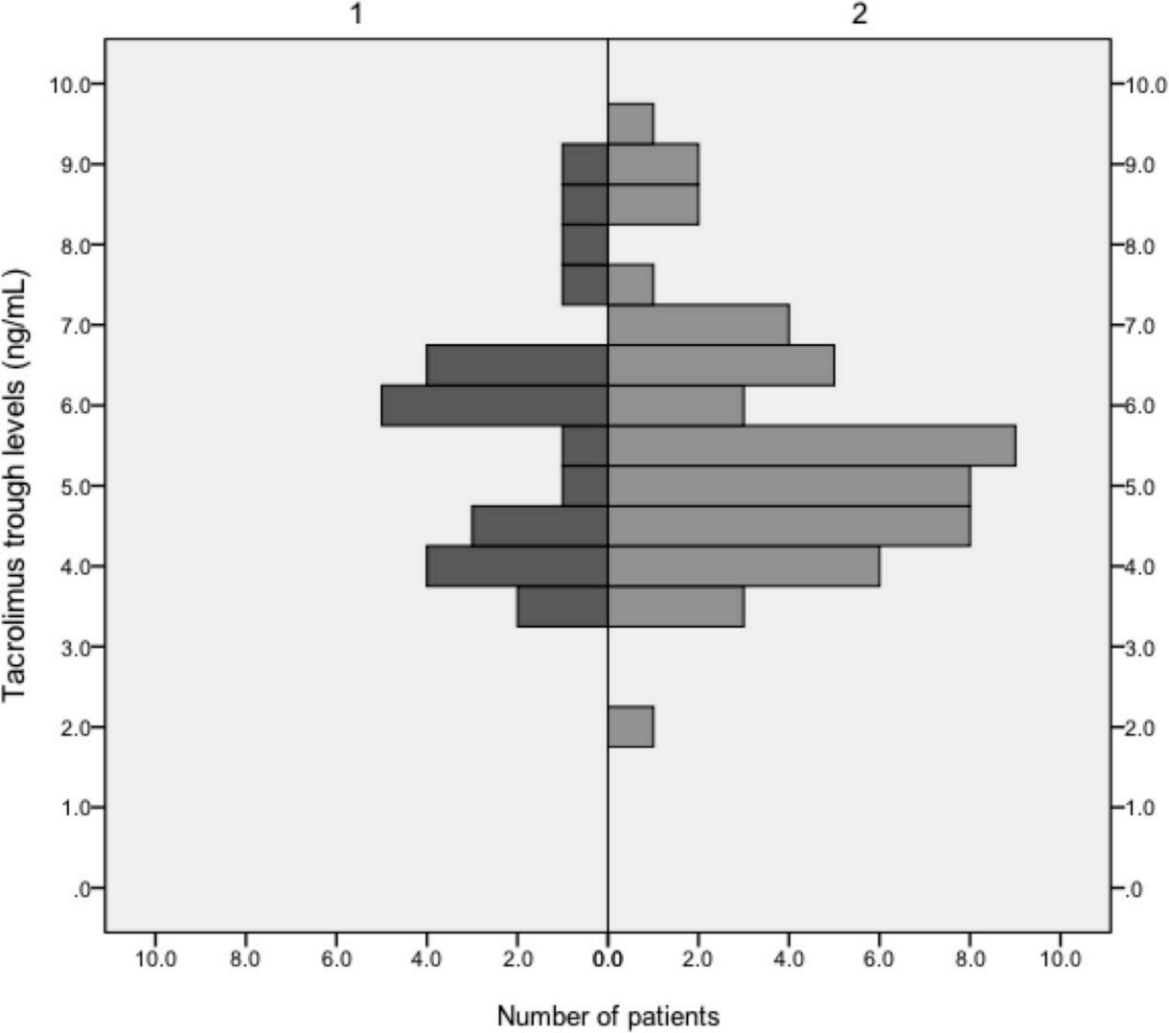
Median tacrolimus trough levels of the 77 patients converted from ciclosporin and immediate release tacrolimus to modified release tacrolimus at the end of the study (12 months after conversion). 1/dark bars = conversion from ciclosporin-based immunosuppression (n = 24), 2/light bars = conversion from tacrolimus-based immunosuppression (n = 53). Please note that scale of ordinate and abscissa differs from Fig 2.

**Fig 4:**
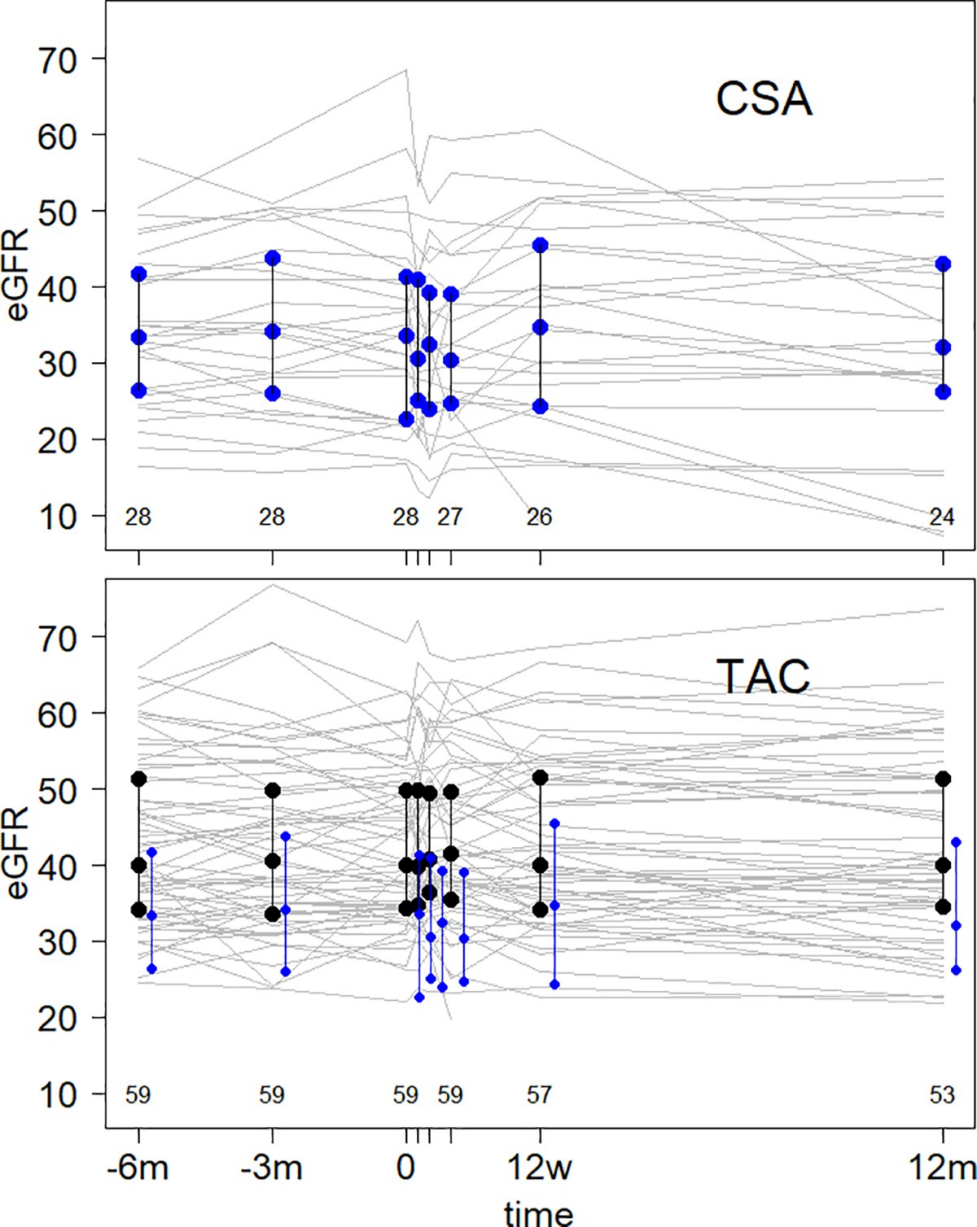
Trajectories of kidney graft function in 87 kidney transplant recipients before and during the first year after conversion from CSA (n = 28) and TAC (n = 59) to TAC MR 4 (median and interquartile range). CSA = ciclosporin A, TAC = tacrolimus, TAC MR4 = tacrolimus modified release, eGFR = estimated glomerular filtration rate, m = month, w = week. The upper field depicts the trajectories in the CSA group (median and interquartile range; blue dots and lines), in the lower field these are superimposed on the trajectories in the TAC group (median and interquartile range; black dots and lines). The x-axis denominates the points in time and the number of compiled eGFR-measurements (in weeks 1 and 2 omitted due to shortage of space. Week 1: CSA n = 25, TAC = 55. Week 2: CSA n = 27, TAC n = 57).

As observed previously, the distribution of *ABCB1* genotypes strongly depends on the population studied. Neither L/D nor C/D ratios exhibited influences of *ABCB1* genotypes on TAC metabolism. The median daily CSA dose at conversion to TAC MR4 was 150 (50–200) mg, and the median daily TAC MR4 dose was 4 (1–10) mg 4 weeks and 3.5 (1–10) mg 12 weeks after conversion, which translates to a conversion rate of roughly 1:40 (1:37.5 and 1:42, respectively). After conversion from CSA to TAC MR4, median TAC MR4 trough levels were 13.8 (7.1–18.9) ng/mL at week 1, 9.5 (8–11.4) ng/mL at week 2, 5.9 (5.4–7.8) ng/mL at week 4, 6.9 (5.6–9.4) ng/mL at month 3 and 5.9 (4.3–6.5) ng/mL at month 12. In patients converted from TAC to TAC MR4 median TAC MR4 trough levels were 4.8 (3.6–6.7) ng/mL at week 1, 5.3 (4.3–6.4) at week 2, 5.2 (4.3–6.6) at week 4, 5.5 (4.6–6.7) ng/mL at month 3 and 5.3 (4.3–6.3) at month 12. In patients converted from CSA to TAC MR4 the median daily dose was 7 (7–9) mg at week 1, 6 (4–7) mg at week 2, 4 (2–7) mg at week 4, 3.5 (2–7) mg at month 3, and 3 (2–5) mg at week 12. In patients converted from TAC to TAC MR4 the median daily dose was 3 (2–5) mg from week 1 to month 12. Figs 2 and 3 illustrate the distribution of TAC trough levels in the first week after conversion in the ITT population, and the median TAC trough levels of all patients who finished the study at 12 months after conversion. In the original PEP study we also could not find influences of *ABCB1* genotypes on TAC metabolism except for level to dose ratios in the *ABCB1* 1236C>T genotype at a single time point (week 2: 0.02599 [CC] vs. 0.05704 [CT] vs. 0.03218 [TT]; p = 0.024), which most likely is of no clinical significance. The observed distribution of genotypes *ABCB1* 1236C>T, 2677G>T/A and 3435C>T in 87 KTR converted from either TAC or CSA to TAC MR4 was not consistent with the Hardy-Weinberg Equilibrium (χ^2^ = 83.29, p < 0.0001; χ^2^ = 21.22, p < 0.0007; χ^2^ = 64.03, p < 0.0001) (Table 4). Fig 4 shows eGFR trajectories of both conversion groups from -6 months to the end of the study.

**Table 4:** Distribution of *ABCB1* genotypes, level to dose and concentration to dose ratios after 12 months of exposure with tacrolimus modified release (n = 87).

*ABCB1 Genotypes*	Distribution	L/D ratio (ng/mL)/(mg/d)	C/D ratio (mg/kg)/d
***3435C>T***			
*CC*	22 (25%)	1.5 (1–2.4)	105.5 (74.2–187.5)
*CT*	5 (6%)	2.85 (2.85–2.85)	208.5 (208.5–208.5)
*TT*	60 (69%)	1.98 (1.08–2.85)	141.3 (78.7–207)
***2677G>T/A***			
*AA*	2 (2%)	1.09 (1.05–1.14)	70.44 (66.72–74.16)
*GA*	0	0	0
*GG*	56 (64%)	1.73 (1.03–2.73)	140.7 (70.52–213.2)
*GT*	17 (20%)	1.77 (1.14–2.3)	127.8 (80–207)
*TA*	1 (1%)	0.75 (0.75–0.75)	52.5 (52.5–52.5)
*TT*	11 (13%)	2.25 (1.74–2.53)	177 (128.8–189.7)
***1236C>T***			
*CC*	38 (44%)	1.34 (0.83–2.05)	91.8 (63.3–180.4)
*CT*	1 (1%)	2.56 (1.9–2.8)	208.5 (86.9–215.4)
*TT*	48 (55%)	1.94 (1.11–2.63)	140.7 (78.7–203)

L/D = level-to-dose, C/D = concentration-to-dose. Data are expressed as frequency and percentage and as median and interquartile range, where appropriate.

## Discussion

In this open label prospective extension study of a randomized controlled trial we converted 87 KTR from either CSA or TAC to TAC MR4, with a follow up of up to 12 months. We investigated the influence of TAC MR4 on intermediate- to long-term renal graft function, and side effects. We defined the distribution of *ABCB1* genotypes in this population and clarified correlations of *ABCB1* 1236C>T, *ABCB1* 2677G>T/A and *ABCB1* 3435C>T genotypes with indicators of TAC MR4 metabolism. Also, we investigated TAC MR4 dose requirements after conversion from CSA and TAC. We found that in stable long-term KTR, the conversion from CSA to TAC MR4 with 0.1 mg per kg BW of TAC, as suggested by the manufacturer, resulted in supra-therapeutic TAC trough levels in a significant proportion of patients. Despite close monitoring and accurate dose adjustments TAC C_0_ levels were above target for more than 12 weeks after conversion. KTR converted from TAC to TAC MR4 in a 1:1 ratio established low trough levels, similar to previous findings [10, 44]. Conversely, retrospective data from 55 KTR demonstrated a 30% increase of the daily TAC dose. More importantly, after conversion from TAC to TAC MR4, trough levels were reported to be lower [45–47]. *De novo* KTR needed as much as 50% more TAC MR4 to attain target trough levels compared to *de novo* TAC [48]. TAC requirements were shown to increase after conversion to TAC MR4 [19, 49]. A retrospective analysis in *de novo* KTR converted to TAC MR4 from TAC within the immediate post-transplant period showed that TAC C_0_ levels remained unchanged in 33%, and doses increased in 47%, and decreased in 20% [50]. The eGFR was reported to remain unchanged after conversion from TAC to TAC MR4 [19, 51], conversely other studies found an increased eGFR with TAC MR4 [44, 52, 53]. A *per protocol* analysis of the conversion from CSA to TAC MR4 in KTR showed no difference of graft function after 24 weeks [54]. In our study, the graft function of KTR converted from CSA to TAC MR4 declined significantly over 12 months, whereas in KTR converted from TAC to TAC MR4 the graft function remained unchanged. In the preceding PEP study, the eGFR in the CSA-based group was 46.7 (40.1–61.8) mL/min at baseline, and 47.6 (36.3–60.8) mL/min at month 24, and in the TAC-based group 45.8 (37.7–55.5) mL/min at baseline, and 47.6 (38.6–60.8) mL/min at month 24 (no significant differences). At the time of initiation of the extension study and conversion to TAC MR4, the eGFR in the CSA group was already lower compared to the eGFR in the TAC group. This may partially explain the differences between the groups. However, the majority of patients from the CSA group experienced supra-therapeutic trough levels after a bodyweight adapted dosing regimen, and stable doses and trough levels in some were achieved only well after three weeks. Thus, CNI toxicity may have contributed to the decline in GFR, which was not observed in those converted from TAC to TAC MR4. Also, those patients had well adjusted, and rather low, median trough levels.

A retrospective observation in 49 KTR with *de novo* TAC MR4 reported almost double dose requirements to achieve trough levels comparable with 30 KTR and TAC [55]. By contrast, doses decreased in *de novo* TAC MR4 KTR [56]. Additional factors may explain the inter-individual variability of TAC metabolism, which is known to be influenced by mutations in the cytochrome-p 450 (CYP) 3A4 [57] and 3A5*1/*3 systems [6, 58], and co-administration of more than 10 mg/d PRED produces a greater demand for TAC/TAC MR4 to achieve target trough levels [59]. Data on the influence of bodyweight on TAC exposure are inconsistent [59, 60]. A model of patient outcomes and treatment costs over a 5-year horizon estimated patient- and graft survival as well as cost-effectiveness favoring TAC MR4 [61] based on the assumption that TAC MR4 would lead to a reduced incidence of acute rejection episodes, thus cutting down expenses for further treatments and consecutive morbidity. This assumption may not hold, however, as acute rejection rates as high as 8% at 6 months and 10% at 12 months after transplantation were observed in patients receiving TAC MR4, compared to 4% and 7.5% in those receiving TAC, respectively, with the necessity for anti-rejection therapy in 18.2% (TAC MR4) and 11.8% (TAC) [11]. Other conversion studies did not observe biopsy proven rejections [56]. The C/D ratios after conversion from TAC to TAC MR4 were observed to remain unchanged, despite a significant reduction of TAC trough levels [16]. *ABCB1* single nucleotide polymorphisms influence CNI metabolism and C/D ratios. Therefore, to uncover the potential influences on TAC metabolism, we examined the distribution of *ABCB1* genotypes 2677G>T/A, 1236C>T, and 3435C>T among the study cohort. Although there are many studies of the effects of *ABCB1* 2677G>T/A [26, 27] [28, 29], 1236C>T [28, 29], and 3435C>T [30–34] on CSA and TAC metabolism, there is insufficient data for TAC MR4. No influences of the *ABCB1* 3435C>T genotype on TAC MR4 levels were demonstrated in Japanese *de novo* KTR [6]. In this study, as well as in the preceding RCT [35], we could not find clinically relevant associations of *ABCB1* 1236C>T, 2677G>T/A and 3435C>T genotypes with TAC MR4 trough levels, although these results are limited by the relatively small sample size. Most deviations from the Hardy-Weinberg Equilibrium indicate either methodological or technical errors [62]. For this study we applied a rare quality criterion and confirmed all mutant homozygous genotypes by repeated analysis of DNA samples. Moreover, the accuracy of the PCR-based genotyping assay was confirmed by direct nucleotide sequence analysis of heterozygous samples. Hence, the HWE-deviation observed in this study may be explained by the relatively small sample size and/or patient selection. It has been proposed that non-adherence would be observed less frequently with a once-daily regimen. This might be the case in some patients. Admittedly, skipping one TAC BID dose reduces the exposure by 50%, whereas one skipped TAC MR4 QD dose reduces this by 100% [63]. Non-adherence to the QD regimen was described as high as 62%, and 40% for the BID regimen [64]. We neither systematically surveyed the adherence nor patient satisfaction. However, we frequently asked whether QD was preferred over BID. Patients with little medication tended to appreciate QD rather than BID, whereas patients who were prescribed large amounts of medication tended to have a neutral position. Many KTR suffer from gastrointestinal symptoms, mostly attributable to chronic renal insufficiency, CNI- and MMF therapy. Conversion to TAC MR4 has been demonstrated to reduce gastrointestinal side effects [65]. We found that adverse events were common, especially gastrointestinal symptoms occurred. A third of those converted from CSA/TAC to TAC MR4 experienced diarrhea, and 3 patients requested to be re-converted to TAC and CSA, respectively, due to intolerable gastrointestinal symptoms. Infections occurred almost three times more often after conversion from TAC to TAC MR4, compared to conversion from CSA. Pruritus was only observed in those converted from CSA. Erroneous intake occurred more frequently in those converted from TAC to TAC MR4. However, incidents and errors with intake or prescriptions were quite common. Major limitations of this study certainly are the non-randomized character of an extension study, as well as the lack of a control group. However, this is somehow counterbalanced by the fact that the original trial was a RCT. Secondly, the study population represents a convenience sample without formal power analyses. Therefore, these results cannot be generalized. Also, there is no information on graft biopsy prior to enrolment with main histologic findings, presence or occurrence of anti-HLA antibodies. We did not conduct surveillance biopsies.

## Conclusions

We found, that in stable long-term kidney transplant recipients, conversion from twice daily TAC to once daily TAC is feasible. We observed supra-therapeutic TAC concentrations in a large portion of KTR after conversion from CSA to once TAC MR4, despite closely monitored TAC trough levels and frequent dose adaptations. If a conversion from CSA to TAC MR4 is considered, we suggest a conversion rate of 1:40 for a rough estimation of TAC MR4 target doses to avoid accidental overdoses.

## Supporting information

S1 File Amendment to the ethics commission of the medical university of Vienna (german). Amendment to the Ethics Commission of the Medical University of Vienna to extend the original randomized controlled trial PEP as PEP-X. Original version (german).
(DOC)

S2 File English translation of the amendment to the ethics commission of the medical university of Vienna drafted in german language. Amendment to the Ethics Commission of the Medical University of Vienna to extend the original randomized controlled trial PEP as PEP-X. Translated version (english).
(DOCX)

S3 File TREND statement. Transparent Reporting of Evaluations with Nonrandomized Designs (TREND) statement checklist.
(PDF)
