# Supplementary material for: A non-randomized trial of conversion from ciclosporin and tacrolimus to tacrolimus MR4 in stable long-term kidney transplant recipients: Graft function and influences of ABCB1 genotypes
Source: PLoS One. 2019 Jul 2;14(7):e0218709. doi: 10.1371/journal.pone.0218709 (PMC6606311; doi:10.1371/journal.pone.0218709)
Supplement: S1 File — Amendment to the Ethics Commission of the Medical University of Vienna to extend the original randomized controlled trial PEP as PEP-X. Original version (german). (DOC) [file pone.0218709.s001.doc]

EK der Medizinischen Universität Wien

Adresse der Ethikkommission (optional)

**Meldung**

Formular für die Meldung von Amendments, schwerwiegenden unerwünschten Ereignissen
und für sonstige Mitteilungen an österreichische Ethik-Kommissionen.

**Version 5.0 vom 03.08.2004 Bitte immer die *aktuelle* Version verwenden (http://ethikkommissionen.at)!**

Borschkegasse 8b

1090 Wien

**MITTEILUNG**

Raum für Eingangsstempel, etc. Bitte Freilassen!

an folgende österreichische Ethik-Kommissionen:

| Ethik-Kommission | EK-Nummer | Datum des Votums | Prüfer |
| --- | --- | --- | --- |
| **Medizinische Universität Wien** | **393/2004** | **22.12.04** | **a.o. Univ. Prof. Dr. Gere Sunder-Plassmann** |
|  |  |  |  |
|  |  |  |  |
|  |  |  |  |
|  |  |  |  |
|  |  |  |  |
|  |  |  |  |

**1.1 MELDUNG einer PROTOKOLLÄNDERUNG (AMENDMENT)**

Begründen Sie die vorgenommenen Protokolländerungen.Legen Sie ein Exemplar, sowie eine Liste der vorgenommenen Änderungen bei.
Machen Sie exakte Angaben über die Bezeichnung des Amendments (falls erforderlich).

**1.2 MELDUNG eines schwerwiegenden unerwünschten Ereignisses bzw. einer mutmasslichen unerwarteten schwerwiegenden nebenwirkung**

Ergeben sich daraus für Ihr Projekt Konsequenzen? Geben Sie eine Stellungnahme dazu ab!
Legen Sie eine Kopie der Meldung an das für Gesundheit zuständige Ministerium bei (wenn zutreffend).

**1.3 SONSTIGE MITTEILUNG**

**2. Angaben zum Forschungsprojekt (EK-Nummer bitte in die obige Tabelle eintragen):**

2.1 Projekttitel (Kurzbezeichnung):

**The Vienna Prograf and Endothelial Cell Progenitor Study - The Vienna PEP-Study**

2.2 Protokollnummer (falls vorhanden): **1.05**

3. Begründung/Stellungnahme/Mitteilung:

| Das Amendment bezieht sich auf die Umstellung der Immunsuppression bei Teilnehmern der klinischen Prüfung "The Vienna Prograf and Endothelial Progenitor Cell Study - The Vienna PEP-Study" am Ende der Studie nach 2 Jahren.  Ziel der Umstellung ist die Untersuchung der Auswirkungen des MDR1/CYP450 Genotyps auf die Talblutspiegel von Tacrolimus mit modifizierter Galenik (Tacrolimus MR4). Es werden Analysen der Auswirkungen des Genotyps auf die concentration/dose ratio durchgeführt werden, was auch Untersuchungen auf vorhandene Polymorphismen im multi-drug resistance transporter 1 (MDR1) Gen (Gensymbol: ABCB1; siehe erstes Amendment vom Juni 2006) sowie im Cytochrom p-450 System (CYP450) beinhalten wird. Die Einwilligung der Patienten zur Umstellung der immunsuppressiven Therapie von Tacrolimus (Prograf) beziehungsweise von Cyclosporin A (Sandimmun Neoral) auf Tacrolimus MR4 (Advagraf) wird eingeholt, das Formular liegt im Anhang bei.  Nach Beendigung der 24 monatigen Studienteilnahme und Einholung der Einverständniserklärung zur Umstellung der immunsuppressiven Therapie von entweder Tacrolimus (Prograf®) oder Ciclosporin A (Sandimmun Neoral®) auf Tacrolimus modified release (Tacrolimus MR4 = FK 506E = Advagraf®) wird diese entsprechend den Angaben des Herstellers (Astellas Pharma GesmbH, Neumarkter Strasse 61, D-81673 München beziehungsweise Linzer Strasse 221, A-1140 Wien) bei Patienten unter Therapie mit Neoral mit initial 0.1 - 0.12 mg Tacrolimus MR4 pro kg Körpergewicht pro Tag mit morgendlicher oraler Gabe erfolgen. Bei Patienten die bereits mit Prograf behndelt weden wird die Umstellung auf Advagraf im Verhältnis 1:1 durchgeführt.  Die Angaben zur Dosierung bei Umstellung von Ciclosporin A (für die Teilnehmer in der Kontrollgruppe der Vienna PEP Study zutreffend) auf Tacrolimus MR4 stammen von den Dokumenten der Homepage der European Medicines Agency (http://www.emea.europa.eu/index/indexh1.htm; abgerufen am 26.09.2007), jene zur Sicherheit und Äquivalenz der erreichten areas under the curve (AUC) beider Tacrolimus-Formulationen aus Alloway et al. (Transplant Proc 2005;37:867-870), die jeweiligen Dokumente können der EK auf Wunsch übermittelt werden.  Die Umstellung der Therapie wird durch a.o. Univ. Prof. Dr. Gere Sunder-Plassmann beziehungsweise durch Dr. Markus Riegersperger durchgeführt werden, beide Klinische Abteilung für Nephrologie und Dialyse, Univ. Klinik für Innere Medizin III, Medizinische Universität Wien, Währinger Gürtel 18-20, 1090 Wien. Der angestrebte Talspiegel wird entsprechend den an unserem Zentrum üblichen klinischen Standards bei Langzeittransplantierten mit 4.0 - 8.0 ng/mL angesetzt, die Spiegelkontrollen werden an der Nephrologischen Spezialambulanz für Nierentransplantierte der Klin. Abt. für Nephrologie und Dialyse, Univ. Klin. für Innere Medizin III, nach folgendem Schema durchgeführt werden: 1 Woche nach Umstellung, 2 Wochen nach Umstellung, 4 Wochen nach Umstellung und 12 Wochen nach Umstellung. |
| --- |

**4. Unterschrift**

|  | Unterschrift des Prüfers |  | Datum | **Datum** |
| --- | --- | --- | --- | --- |
