# Supplementary material for: A non-randomized trial of conversion from ciclosporin and tacrolimus to tacrolimus MR4 in stable long-term kidney transplant recipients: Graft function and influences of ABCB1 genotypes
Source: PLoS One. 2019 Jul 2;14(7):e0218709. doi: 10.1371/journal.pone.0218709 (PMC6606311; doi:10.1371/journal.pone.0218709)
Supplement: S2 File — Amendment to the Ethics Commission of the Medical University of Vienna to extend the original randomized controlled trial PEP as PEP-X. Translated version (english). (DOCX) [file pone.0218709.s002.docx]

**English translation of the amendment to the Ethics Commission of the Medical University of Vienna drafted in german language.**

The amendment refers to the conversion of immunosuppressive agents among participants in the clinical trial "The Vienna Prograf and Endothelial Progenitor Cell Study - The Vienna PEP Study" at the end of the study after 2 years in the extension study PEP-X.

The aim of the conversion is to investigate the effects of the MDR1/CYP450 genotype on the trough blood levels of tacrolimus with modified galenics (tacrolimus MR4; Advagraf®). Analyzes of the effects of the genotype on the concentration/dose ratio will be carried out, as well as studies on existing polymorphisms in the multi-drug resistance transporter 1 (MDR1) gene (gene symbol: *ABCB1*, see first amendment of June 2006) and in the cytochrome p- 450 system (CYP450). The written informed consent to conversion of either tacrolimus (Prograf®) or cyclosporin A (Sandimmun Neoral®) to tacrolimus MR4 will be obtained, the form is enclosed.

Upon completion of 24 months of study participation of the PEP-study, and consent to convert the immunosuppressant therapy of either tacrolimus or ciclosporin A to tacrolimus modified-release, it will be used as indicated (Astellas Pharma GesmbH, Neumarkter Strasse 61, D-81673 Munich or Linzer Strasse 221, A-1140 Vienna). In patients treated with ciclosporin A, the initial dose will be 0.1 - 0.12 mg tacrolimus MR4 per kg of body weight per day with oral morning administration. In patients who are already treated with Prograf, the conversion to Advagraf will be performed in a 1:1 ratio.

The dosage data for the conversion of cyclosporin A to tacrolimus MR4 are taken from the documents of the European Medicines Agency homepage (http://www.emea.europa.eu/index/ indexh1.htm, retrieved 26.09.2007), those on the safety and equivalence of the achieved areas under the curve (AUC) of both tacrolimus formulations from Alloway et al. (Transplant Proc 2005; 37: 867-870), the respective documents can be sent on request.

The conversion will be performed by Univ. Prof. Dr. Gere Sunder-Plassmann, and Dr. Markus Riegersperger, respectively, both Division of Nephrology and Dialysis, Department of Medicine III, Medical University of Vienna, Währinger Gürtel 18-20, 1090 Vienna. The desired trough level is set at 4.0-8.0 ng/mL according to the usual clinical standards for long-term transplant patients at our center. The trough level analyses are performed at the Medical University of Vienna, according to the following schedule: 1 week after conversion, 2 weeks after conversion, 4 weeks after conversion, 12 weeks and 12 months after conversion.
